# Hyperglycemia triggers HIPK2 protein degradation

**DOI:** 10.18632/oncotarget.13595

**Published:** 2016-11-25

**Authors:** Silvia Baldari, Alessia Garufi, Marisa Granato, Laura Cuomo, Giuseppa Pistritto, Mara Cirone, Gabriella D'Orazi

**Affiliations:** ^1^ Department of Research, Advanced Diagnostics, and Technological Innovation, Regina Elena National Cancer Institute, 00144 Rome, Italy; ^2^ Department of Medical Sciences, Tumor Biology Unit, University ‘G. d'Annunzio’, 66013 Chieti, Italy; ^3^ Department of Experimental Medicine, Pasteur-Fondazione Cenci Bolognetti Institute, Sapienza University, 00100 Rome, Italy; ^4^ U.O.C. Clinical Pathology, A.C.O., San Filippo Neri Hospital, 00100 Rome, Italy; ^5^ Department of Systems Medicine, University Tor Vergata, 00133 Rome, Italy

**Keywords:** HIPK2, cancer, hyperglycemia, p53, PP2A

## Abstract

Homeodomain interacting protein kinase-2 (HIPK2) is an evolutionary conserved kinase that modulates several key molecular pathways to restrain tumor growth and induce p53-depending apoptotic cell-death in response to anticancer therapies. HIPK2 silencing in cancer cells leads to chemoresistance and cancer progression, in part due to p53 inhibition. Recently, hyperglycemia has been shown to reduce p53 phosphorylation at serine 46 (Ser46), the target residue of HIPK2, thus impairing p53 apoptotic function. Here we asked whether hyperglycemia could, upstream of p53, target HIPK2. We focused on the effect of high glucose (HG) on HIPK2 protein stability and the underlying mechanisms. We found that HG reduced HIPK2 protein levels, therefore impairing HIPK2-induced p53 apoptotic activity. HG-triggered HIPK2 protein downregulation was rescued by both proteasome inhibitor MG132 and by protein phosphatase inhibitors Calyculin A (CL-A) and Okadaic Acid (OA). Looking for the phosphatase involved, we found that protein phosphatase 2A (PP2A) induced HIPK2 degradation, as evidenced by directly activating PP2A with FTY720 or by silencing PP2A with siRNA in HG condition. The effect of PP2A on HIPK2 protein degradation could be in part due to hypoxia-inducible factor-1 (HIF-1) activity which has been previously shown to induce HIPK2 proteasomal degradation through several ubiquitin ligases. Validation analysed performed with HIF-1α dominant negative or with silencing of Siah2 ubiquitin ligase clearly showed rescue of HG-induced HIPK2 degradation. These findings demonstrate how hyperglycemia, through a complex protein cascade, induced HIPK2 downregulation and consequently impaired p53 apoptotic activity, revealing a novel link between diabetes/obesity and tumor resistance to therapies.

## INTRODUCTION

Homeodomain interacting protein kinase 2 (HIPK2) is a nuclear serine/threonine kinase that is considered a central switch in driving cancer cells toward apoptotic cell death upon genotoxic stress [1]. In response to chemotherapeutic drugs or to UV and ionizing irradiation HIPK2 is activated to phosphorylate key molecules such as oncosuppressor tumour suppressor p53 at serine 46 (Ser46) [2, 3], the anti-apoptotic co-repressor CtBP [4], the p53 inhibitor MDM2 [5], and the prosurvival dominant negative isoform of the p53 family member p63 (ΔNp63α) [6], leading to apoptosis. Inhibition of HIPK2 has been shown to have a negative impact on both p53 function and tumor response to therapies [7]. Previous studies reported that integrin alpha(6)beta(4) overexpression correlates with HIPK2 cytoplasmic localization in breast cancer, leading to impairment of p53 apoptotic activity [8]. Other studies showed that Src kinase suppresses the apoptotic p53 pathway by HIPK2 phosphorylation and relocalization to the cytoplasm [9]. Few HIPK2 mutations have been found in human acute myeloid leukemias (AML), leading to aberrant HIPK2 nuclear distribution with impairment of p53 apoptotic activity [10]. Other studies found a link between oncogene E6 of genital high-risk human papillomavirus (HPV) and HIPK2. The authors found that E6 of beta2PV types (HPV23 and HPV38), physically interacts with HIPK2 enforcing dissociation of the HIPK2/p53 complex and consequently inhibiting HIPK2-mediated p53Ser46 phosphorylation [11]. Moreover, multiple lines of evidence showed that the HIPK2 protein levels may be regulated by several E3 ubiquitin ligases under both normal conditions or external stimuli, according to cellular needs. In unstressed conditions, HIPK2 is degraded by the RING family ligase seven in absentia homolog-1 (Siah1) while, in response to DNA damage, the HIPK2/Siah1 complex is disrupted by the ATM/ATR-dependent phosphorylation of Siah1 resulting in HIPK2 stabilization and activation [12, 13]. An intriguing regulatory circuitry between MDM2 and HIPK2/p53 axis revealed that sublethal DNA damage induces HIPK2 inhibition by a protein degradation mechanism involving p53-induced MDM2 activity [14]. On the other hand, severe DNA damage activates HIPK2 that, by phosphorylating MDM2 for proteasomal degradation [15], may overcome the MDM2-induced p53 inactivation restoring p53 apoptotic activity [5]. Other E3 ubiquitin ligases involved in HIPK2 degradation are induced by hypoxia, a hallmark of tumor progression and failure of tumor therapies [16]. The key mediator in response to decreased oxygen availability is the transcription factor hypoxia-inducible factor-1 (HIF-1) that consists of the constitutively expressed HIF-1β subunit and the HIF-1α subunit, whose stability is stimulated by low oxygen or by genetic alterations [16]. Thus, independently of the oxygen conditions, HIF-1α may be upregulated by transcriptional and translational mechanisms and therefore have a broad impact on the expression of many genes, induced by HIF-1 activity, involved in cell proliferation, chemoresistance, motility, and apoptosis (i.e., *VEGF*, *MDR1*, *GLUT-1*) [17, 18]. A novel HIF-1 target gene, upregulated upon hypoxia, is WD40-repeat/SOCS box protein *WSB-1* [19, 20] that has been shown to induce HIPK2 degradation [21]. Moreover, under hypoxia, the RING family ligase Siah2 is also activated to increase HIPK2/Siah2 interaction, by a still unknown mechanism, that induces HIPK2 degradation [22].

We previously showed that high glucose (HG) reduces p53 phosphorylation at Ser46 that can be rescued by the use of Calyculin A (CL-A) [23], a cell-permeable phosphatase inhibitor which has been shown to inhibit protein phosphatase A2 (PP2A) and therefore enhance ionizing radiation-induced p53Ser46 phosphorylation [24]. Here we wanted to evaluate whether HG could target HIPK2, upstream of p53. The rationale was dictated not only by the finding that HG induces p53Ser46 inactivation in part through PP2A, but also by findings showing that hyperglycemia increases *HIF-1α* gene transcription [25] and induces HIF-1-regulated genes, irrespective of oxygen levels [26].

## RESULTS

### High glucose (HG) reduces HIPK2 protein levels in cancer cells

**Figure 1:**
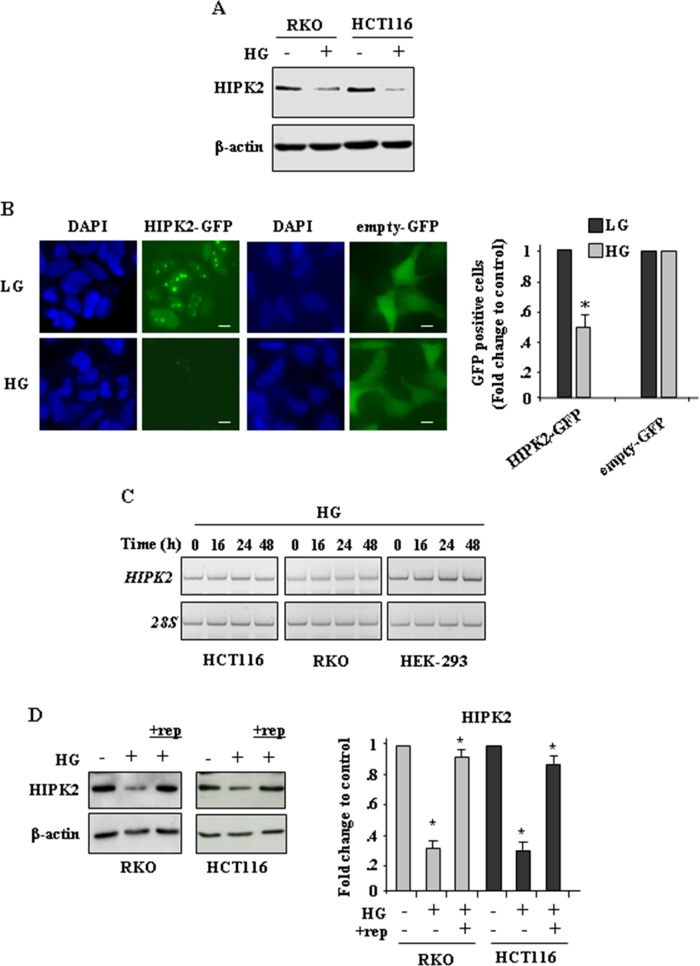
High glucose (HG) reduces HIPK2 protein levels in cancer cells **A.** Western blot analysis of endogenous HIPK2 protein levels in RKO and HCT116 cells cultured in LG (HG-) or HG for 24 h. Anti-β-actin was used as protein loading control. **B, left panel.** Immunofluorescence analysis of HEK-293 cells transfected with HIPK2-GFP or empty-GFP vectors and cultured in LG or HG condition for 24 h. Nuclei were stained with DAPI. Bars, 10 μm. **B, right panel**. Analysis of GFP-positive cells was performed by visualizing at least 200 DAPI-positive cells/group (HIPK2-GFP and empty-GFP) and quantified with respect to control (LG condition) set to 1.0. *P < 0.001. **C.** RKO, HCT116 and HEK-293 cells were grown in high glucose (HG) condition for 16, 24 and 48 h before being assayed for semi-quantitative RT-PCR of HIPK2 mRNA. 28S was used as a control for efficiency of RNA extraction and transcription. **D.** Western blot analysis of endogenous HIPK2 protein levels in RKO and HCT116 cells cultured in LG (HG-) or HG (HG+) for 24 h and after switching back the HG sample to LG condition (+rep -replenishment) for 24 h. Anti-β-actin was used as protein loading control. One representative experiment is shown. Data of relative quantification of HIPK2 levels are presented in the right panel as mean ± S:E.M. (*n* = 4) (one way ANOVA plus Bonferroni correction). *P<0.001 (HG *versus* ctr and +rep *versus* HG).

To evaluate the effect of hyperglicemia on HIPK2 expression, RKO and HCT116 cells were cultured in medium with low glucose (LG) or with high-glucose (HG), as previously reported [23, 27] (see Methods). The results show that HG markedly reduced HIPK2 protein levels (Figure 1A). To assess whether HG could affect HIPK2 cellular localization, HIPK2-GFP protein was overexpressed in HEK-293 cells in dose and time conditions that did not modify cell viability and, twenty-four hours after transfection, cells were transferred in LG and HG conditions. Analysis of the green fluorescent proteins show that HG turned specifically off the HIPK2-GFP signal, compared to the empty-GFP-signal (Figure 1B, left panel), as also evidenced in the plotted graph (Figure 1B, right panel), suggesting that HG could induce protein downregulation rather than cellular delocalization. HG-induced reduction of HIPK2 protein level was not accompanied by changes in *HIPK2* mRNA, as revealed by RT-PCR (Figure 1C). Finally, replenishment (rep.) of HG medium with LG medium efficiently restored HIPK2 protein levels (Figure 1D, compare HG with HG+rep). Collectively, these data show that HG induced a degradative mechanism able to reduce HIPK2 protein levels that could be rescued by switching back cells to LG condition.

### High glucose promotes protein phosphatase A2 (PP2A)-dependent HIPK2 protein degradation

**Figure 2:**
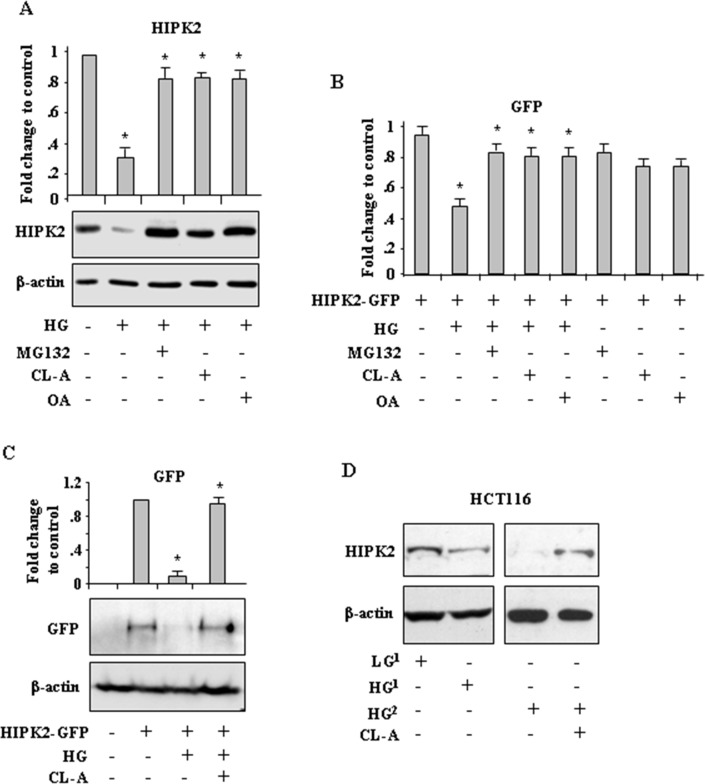
High glucose (HG) promotes phosphatase-dependent HIPK2 protein degradation **A.** Western blot analysis (lower panel) and relative quantification (upper panel) of endogenous HIPK2 protein levels in HCT116 cells cultured in HG with or without MG132, Calyculin A (CL-A) or Okadaic acid (OA). Anti-β-actin was used as protein loading control. A representative image is shown. Data of relative quantification of HIPK2 levels from three different experiments (upper panel) are presented as mean± S.E.M. (*n*=3) and quantified with respect to control set to 1.0. *P < 0.001. (HG *versus* ctr, MG132 *versus* HG, CL-A *versus* HG, OA *versus* HG). **B.** HEK-293 cells were transfected with HIPK2-GFP vector and 24 h after transfection switched in HG condition for 24 h with or without MG132, CL-A, or OA. Analysis of GFP-positive cells was performed by visualizing at least 200 DAPI-positive cells/group and quantified with respect to control (HIPK2-GFP/LG condition) set to 1.0. *P < 0.001. **C.** Western blot analysis (lower panel) and relative quantification (upper panel) of GFP levels in RKO cells transfected with HIPK2-GFP vector and 24 h after transfection switched in HG condition for 24 h with or without CL-A. Anti-β-actin was used as protein loading control. *P < 0.001. (HG *versus* HIPK2-GFP and HG/CL-A *versus* HG/HIPK2-GFP). **D.** Western blot analysis of endogenous HIPK2 protein in HCT116 cells cultured for 24 h in the presence of serum coming from different patients, evidenced as numbers at the height: one normo-glycemic sera (glycemia ≤ 90) (LG^1^) and two hyperglycemic sera (glycemia ≥ 300) derived from patients with type 2 diabetes (DM2) (HG^1^ and HG^2^), with or without CL-A. Anti-β-actin was used as protein loading control.

To investigate the mechanisms of HG-induced HIPK2 downregulation, cells were cultured in HG condition with or without treatment with proteasome inhibitor MG132, phosphatase inhibitor Calyculin A (CL-A) [24], or Okadaic acid (OA), a specific and potent inhibitor of protein phosphatases 2A (PP2A) [28]. As shown in Figure 2A, MG132 as well as CL-A and OA significantly rescued the HG-reduced HIPK2 protein levels. Matching results were obtained by counting the number of HIPK2-GFP-positive cells treated as above (Figure 2B). Moreover, western blot analysis show that the HG-induced HIPK2-GFP degradation was counteracted by CL-A treatment (Figure 2C). As a proof of principle, HIPK2 protein levels were analysed in a more physiologic system. To this aim, cells were cultured in the presence of hyperglycemic sera (HG) derived from patients with type 2 diabetes or in the presence of normo-glycemic sera (NG) derived from healthy donors, as previously reported [29]. The results show that culturing cells with hyperglycemic sera (HG) reduced the HIPK2 levels, compared to the effect obtained culturing cells with normo-glycemic sera (NG) (Figure 2D); in agreement with the above results, CL-A treatment markedly rescued the HIPK2 protein levels reduced by hyperglycemic sera (HG) (Figure 2D).

**Figure 3:**
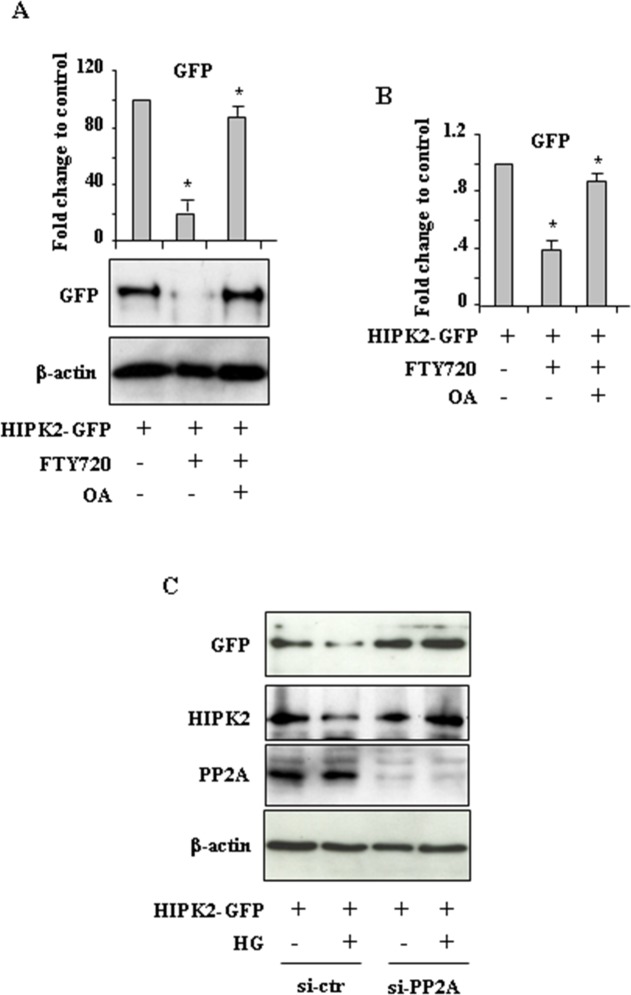
PP2A induces HIPK2 degradation **A.** Western blot analysis and relative quantification of GFP levels in HEK-293 cells transfected with HIPK2-GFP vector and treated with PP2A activator FTY720, with or without Okadaic acid (OA). Data of relative quantification of HIPK2-GFP levels are presented as mean± S.E.M. (*n*=3). *P < 0.001 (FTY720 *versus* HIPK2-GFP and FTY720/OA *versus* FTY720). **B.** HEK-293 cells were transfected with HIPK2-GFP vector and 24 h after transfection treated with PP2A activator FTY720, with or without Okadaic acid (OA). Analysis of GFP-positive cells was performed by visualizing at least 200 DAPI-positive cells/group and quantified with respect to control (HIPK2-GFP/LG condition) set to 1.0. *P < 0.001. The quantification of GFP-positive cells is presented as mean ± S.E.M. (one way ANOVA plus Bonferroni correction). *P<0.001 (FTY720 *versus* HIPK2-GFP and FTY720/OA *versus* FTY720). **C.** HEK-293 cells were transfected with siRNA for PP2a-Cα (si-PP2A) or with control siRNA (si-ctr) and 24 h later transfected with HIPK2-GFP before switching to HG for 24 h. Western blot analysis shows the levels of both exogenous HIPK2-GFP and endogenous HIPK2 protein levels. Anti-β-actin was used as protein loading control.

To determine whether PP2A was involved in HIPK2 degradation we took advantage of FTY720 (Fingolimod, Gilenya), a potent PP2A-activating drug [30]. As shown in Figure 3A, FTY720 markedly reduced HIPK2-GFP levels that were efficiently rescued by OA treatment. In parallel experiments, similar results were obtained by counting the number of HIPK2-GFP-positive cells (Figure 3B). Considering that the chemical antagonists may have offtargets in general and OA and CL-A may confer cellular phenotypes by targeting other phosphatase family members, we carried RNA interference to clarify the specific involvement of PP2A in our setting. The results show that the reduction of both HIPK2-GFP and endogenous HIPK2 levels in HG condition were remarkably impaired by PP2A interference (Figure 3C, compare HG in si-ctr with HG in si-PP2A). Similar results were obtained by using FTY720 (not shown) instead of HG. Interestingly, we noticed that PP2A silencing did not affect HIPK2 stability under normal condition. Altogether, these data demonstrate that HG promoted HIPK2 protein degradation in part depending on PP2A.

### Evaluation of the role of HIF-1α expression in HG-induced HIPK2 degradation

**Figure 4:**
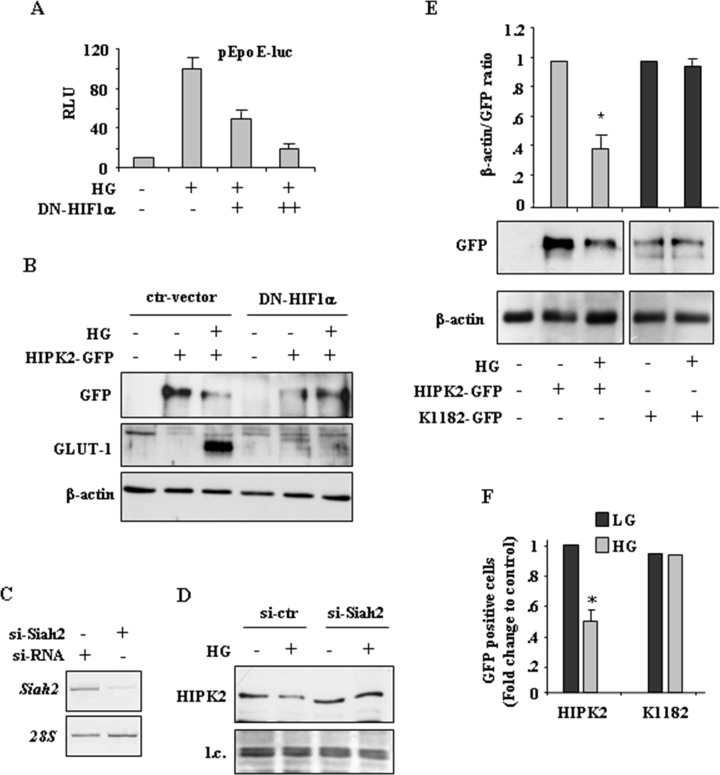
HIF-1 activity is involved in HIPK2 degradation in HG condition **A.** HEK-293 cells were transiently co-transfected with DN-HIF-1α expression vector (1 and 2μg) along with pEpoE-luc reporter (0.1 μg) and 24 h after transfection switched to HG condition for 36 h before luciferase activity was assayed. Relative Luciferase Units (RLU) normalized to β-gal is shown. The shown data represent the mean ± S.D. from three independent experiments performed in duplicate. **B.** HEK293 cells were co-transfected with HIPK2-GFP vector and DN-HIF-1α or control vector (ctr-vector) and 24 h later cultured in HG for 24 h. The expression levels of HIPK2-GFP and GLUT-1 were analysed by western blotting. Anti-β-actin was used as protein loading control. **C.** RKO cells were transfected with si-Siah2 and si-RNA and mRNA expression analysed 48 h later by RT-PCR. **D.** Cells transfected as in (C) were switched to HG condition and endogenous HIPK2 levels analysed by western blot 24 h later. l.c.: loading control. **E.** Western blot analysis (lower panel) and relative quantification (upper panel) of GFP levels in HEK-293 cells transfected with HIPK2-GFP and K1182-GFP expression vectors and 24 h after transfection switched in HG condition for 24 h. Anti-β-actin was used as protein loading control. Data of relative quantification of GFP levels are presented as mean ± S.D. *P < 0.001. **F.** HEK-293 cells transfected with HIPK2-GFP or K1182-GFP expression vectors and cultured in LG or HG condition for 24 h. Analysis of GFP-positive cells was performed by visualizing at least 200 DAPI-positive cells/group (HIPK2-GFP and K1182-GFP) and quantified with respect to control (LG condition) set to 1.0. *P < 0.001.

We previously reported that HG upregulates HIF-1α and induces the expression of some HIF-1 target genes, including *GLUT-1* and *HK2* glycolytic enzyme, that have been shown to protect from drug-induced cancer cell death [25]. As HIF-1α overexpression and HIF-1 activity have been linked to HIPK2 protein degradation [20, 22, 31, 32], we next aimed at evaluating their role in HIPK2 downregulation in HG condition. We first used a transient reporter assay in which endogenous HIF-1 binds to hypoxia responsive elements (HREs) of erythropoietin gene promoter cloned upstream of a luciferase transcriptional reporter (pEpoE-luc) [33, 34]. To underline the role of the HIF-1α subunit in this setting, cells were co-transfected with increasing amount of HIF-1α dominant negative (DN) vector without DNA binding and transactivation domains, as previously reported [35, 36]. As shown in Figure 4A, the HG-induced pEpoE-luc activity was efficiently reduced by DN-HIF-1α co-expression. Next, cells were co-transfected with HIPK2-GFP and DN-HIF-1α expression vectors and, after transfection, switched to HG condition. The results show that the reduction of HIPK2-GFP levels in HG condition was remarkably impaired by DN-HIF-1α co-expression (Figure 4B, compare HG in ctr-vector with HG in DN-HIF-1α). The induction of HIF1 activity in HG was monitored by the expression of HIF1-target GLUT-1 (Figure 4B). Interestingly, as for PP2A silencing (see above), the use of DN-HIF-1α vector did not modify HIPK2 stability under normal condition. Among the E3 ubiquitin ligases involved in hypoxia-induced HIPK2 degradation is Siah2 [22]. Therefore, we carried RNA interference to clarify the specific involvement of Siah2 in our setting. The results show that the reduction of endogenous HIPK2 levels in HG condition was remarkably impaired by Siah2 silencing (Figure 4C, and 4D). Finally, we aimed at evaluating the role of HIPK2^K1182R^ mutant (herein referred as K1182), resistant to proteasomal degradation [14], in HG condition. The results show that the degradation of HIPK2-GFP protein in HG did not affect the K1182 mutant protein (Figure 4E). Matching results were obtained by counting the number of HIPK2-GFP and K1182-GFP fluorescent cells upon LG and HG conditions (Figure 4F). As HIPK2 may be tightly controlled by hierarchically occurring posttranslational modifications (PTM) [37], we also evaluated whether the HG condition could affect HIPK2 phosphorylation. To this aim, overexpressed HIPK2-GFP protein was immunoprecipitated from cells allowed to stand in either LG or HG condition, before undergoing western immunoblotting with anti-phospho-Ser/Thr antibody. The results show that, switching cells from LG to HG conditions, did not modify HIPK2 Ser/Thr phosphorylation (Supplementary Figure S1). Although this technique did not detect modifications of HIPK2 phosphorylation in HG condition, it cannot be excluded that HIPK2 degradation is independent from HG-induced PTM. A more sensitive technique would help, in future studies, to clarify this issue. Altogether, these results suggest that HIF-1α upregulation upon HG was, at least in part, responsible of HIPK2 protein degradation that could be rescued by either inhibiting HIF-1α or ubiquitin ligase Siah2 expression.

### HG impairs HIPK2 apoptotic activity

**Figure 5:**
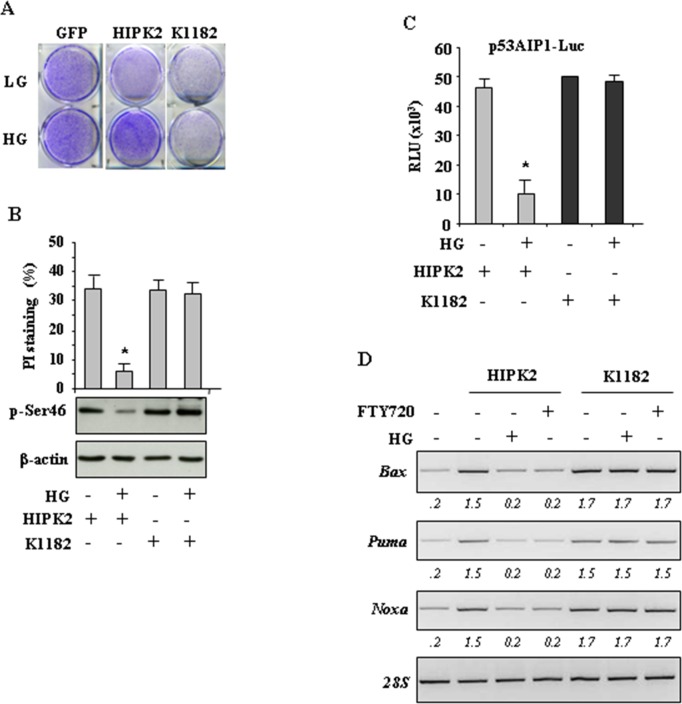
HG impairs HIPK2 apoptotic activity **A.** Clonogenic survival assay of HCT116 transiently transfected with HIPK2-GFP, K1182-GFP and empty-GFP expression vectors. After transfection cells were switched to HG condition and death-resistant cells were stained with crystal violet 1 week later. **B.** RKO cells were transfected with HIPK2-GFP or K1182-GFP expression vectors and 24 h later cultured in HG for 24 h. Twenty-four hours later, cells were in part fixed and stained with PI for subG1 evaluation (upper panel) or lysed and analyzed by western immunoblotting to assess p-Ser46 (lower panel). Data are presented as mean ± S.D. *P < 0.001. **C.** RKO cells were transiently co-transfected with p53AIP1-luc reporter and HIPK2-GFP or K1182-GFP expression vectors. Twenty-four hours after transfection culture medium was changed with HG medium. Results, normalized to β-gal activity are the mean of three independent experiments ± S.D. performed in duplicate. *P = 0.001 **D.** RKO cells were transfected with HIPK2-GFP or K1182-GFP expression vectors and, after transfection, switched to either HG condition for 24 h or FTY720 treatment for 16 h, before being assayed for semi-quantitative RT-PCR of p53 target genes. 28S was used as a control for efficiency of RNA extraction and transcription. Densitometry was performed with ImageJ software and relative band intensity normalized to 28s and quantified with respect to controls set to 1.0.

Finally, the effect of HG on HIPK2 biological activity was analysed. We first performed a long-term survival assay of cells transiently transfected with HIPK2-GFP and K1182-GFP expression vectors. As shown in Figure 5A, the cell survival was reduced by HIPK2 overexpression in normal condition (LG) while it was not affected when cells were switched to HG condition. Interestingly, the effect of K1182 on cell survival was not modified by HG condition, in agreement with the above results that HG did not induce K1182 protein degradation. Next, we performed FACS analysis with propidium iodide (PI) staning to evaluate subG1, apoptotic cell death. As shown in Figure 5B (upper panel), HIPK2 overexpression induced apoptotic cell death that was impaired by switching cells to HG condition. As expected, the K1182-induced apoptosis was not inhibited by HG condition (Figure 5B, upper panel). Of note, Western blotting show that p53Ser46 phosphorylation (p-Ser46), induced by HIPK2, was reduced by HG while p-Ser46 induced by K1182, was not modified by HG (Figure 5B, lower panel). Then, we evaluated the p53 transcriptional activity by luciferase assay. To this aim, cells were co-transfected with the p53 responsive p53AIP-luciferase reporter (target of p53Ser46) along with HIPK2-GFP or K1182-GFP expression vectors. As shown in Figure 5C, the HIPK2-induced p53AIP1-luc activity was remarkably reduced by HG condition, while the K1182-induced p53AIP1-luc activity was not modified by HG condition. (Figure 5C). Finally, the expression of p53-induced apoptotic target genes (i.e., *Bax*, *Noxa* and *Puma*) was evaluated by RT-PCR analysis. As shown in Figure 5D, the HIPK2-induced transcription of apoptotic genes was impaired by both HG condition and FTY720 treatment while, on the contrary, the K1182-induced transcription of p53 apoptotic target genes was not inhibited by HG or FTH720 (Figure 5D), as also evidenced by densitometric analysis (Figure 5D, numbers underneath the images). Altogether, these data indicate that HG strongly impaired HIPK2-induced p53 apoptotic activity. In agreement, a HIPK2 degradation-resistant mutant (K1182) was able to overcome the negative effect of HG and therefore to preserve p53 activity.

## DISCUSSION

**Figure 6:**
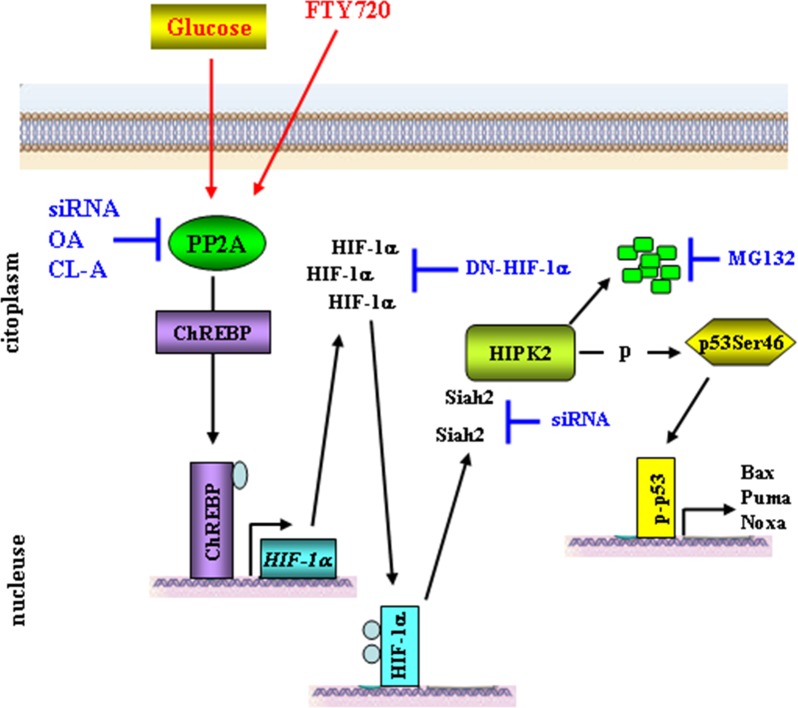
Proposed model for HG-induced HIPK2 degradation High glucose (HG) activates PP2A [51] which in turn dephosphorylates ChREBP allowing its nuclear translocation and binding to the *HIF-1α* promoter [25, 53]. HIF-1α accumulates in the cytoplasm and translocates to the nucleus to bind to the HIF-1β subunit and activate HIF-1-mediated transcription of target gene [17, 18]. Hypoxic condition allows increased HIPK2/Siah2 interaction inducing proteasomal degradation of the kinase [22]. HIPK2 downregulation impairs p53 phosphorylation at Ser46 and reduces apoptotic gene transcription [7]. PP2A activator FTY720 can mimic the HG-induced HIPK2 degradation. HIPK2 stability can be rescued by inhibiting PP2A transcription with siRNA or PP2A activity with CL-A (calyculin A), OA (okadaic acid), by blocking HIF-1α with dominant negative (DN) vector, or by blocking *Siah2* expression by siRNA, finally, HIPK2 levels can be rescued by inhibiting proteasome with MG132 (this manuscript).

HIPK2 is a key regulator of DNA damage-induced apoptosis [1, 38, 39]. In response to DNA damage or chemotherapeutic drugs, activated HIPK2 induces apoptosis via phosphorylation of target proteins such as p53, MDM2, ΔNp63α and CtBP [2–6]. As apoptosis alteration is responsible, among other effects, of tumor resistance to therapies [40], the regulation of HIPK2 expression level and activity is of particular relevance for the determination of cell fate between survival and death. Previous studies established that HIPK2 protein stability is tightly regulated by ubiquitin-proteasome system [12–14, 20–22, 32, 41]. In the present study, we have shown that hyperglycemia promotes the degradation of HIPK2 in parte through ubiquitin ligase Siah2 downstream of a protein cascade including PP2A and HIF-1α, as summarized in the scheme (Figure 6). By regulating HIPK2 protein stability, the metabolic HG condition may have a role in cellular decision-making between survival and apoptosis, in response to anticancer therapies.

Hyperglycemia is defined as a state of excess glucose concentration in the blood, which develops when the body has too little insulin or when the body cannot use insulin properly [42]. Hyperglycemia is a hallmark for diabetes mellitus but other medical condition such as obesity, pancreatitis, chronic stress and cancer can induce it [43–47]. There is evidence that high blood glucose may contribute to cancer progression, apoptosis inhibition, metastasis, and chemotherapy resistance by modifying several molecular pathways [27, 48–50]. In this regard, we previously showed that HG impairs p53 phosphorylation at Ser46 thus reducing p53 apoptotic activity [23]. Interestingly, the use of the PP2A inhibitor Calyculin A restores Ser46 phosphorylation and drug-induce cell death [23]. HG has been shown to activate PP2A [51] and knockdown of PP2A by RNAi is able to enhance ionizing radiation-induced p53Ser46 phosphorylation and apoptosis, suggesting that Ser46 is a target of PP2A activity [24], however, whether the effect is direct on p53 or not has not been clarified. In our study, we added a level of knowledge to this issue by demonstrating that HG-induced HIPK2 degradation strongly inhibited p53 apoptotic activity. The degradation of HIPK2 by HG was dependent on PP2A as elucidated by either PP2A activation with FTY720 [30] or PP2A knockdown with siRNA, thus suggesting that the effect of PP2A on p53Ser46 phosphorylation is not direct but rather mediated by HIPK2 downregulation. As protein degradation is often related to changes in the phosphorylation status, we hypothesized that HIPK2 degradation could be the consequence of PP2A-induced HIPK2 dephosphorylation. However, this was not the case. Although HIPK2-Ser/Thr phosphorylation status did not change under HG condition (Supplementary Figure S1) we do not feel to exclude that these posttranslational modifications may happen under HG and further studies with more sensitive techniques will hopefully allow to clarify this issue.

As previous studies established that HIPK2 protein stability is tightly regulated by several ubiquitin ligases acting in different cellular conditions [12–14, 20–22, 32, 41], we asked what was the link between HG, PP2A and HIPK2 stability. Data from the literature reported that PP2A activation, upon increased glucose flux, induces carbohydrate response element binding protein (ChREBP) dephosphorylation and nuclear translocation to bind to the proximal *HIF1A* promoter, stimulating its transcription and HIF-1α stabilization even in normoxic condition [26, 52–54] (Figure 6). Although we did not assess in this study the levels of ChREBP, in support of that mechanism we previously found that HG increases HIF-1α expression with upregulation of genes target of HIF-1 activity (i.e., *GLUT-1* and *HK2*) [25]. Therefore, as hypoxia and HIF-1 activity have been shown to induce HIPK2 proteasomal degradation through several ubiquitin ligases, including WSB1 and Siah2 [21, 22], we supposed that HIF-1, even in normoxic condition, could be the link between HG condition and HIPK2 degradation. Our hypothesis was supported by data showing that, reducing HIF-1α levels with a dominant negative vector or targeting RNA directed against *Siah2* counteracted HG-induced HIPK2 degradation. *Siah2* knock-down phenocopied *PP2A* knock-down, supporting the notion that the PP2A/HIF-1α/Siah-2 axis was responsible for HIPK2 degradation under HG conditions (Figure 6). However, given that we always detected markedly decreased HIPK2 levels under HG, but never a complete elimination of the kinase, we cannot exclude that other ligases might be involved. Further studies will allow to clarify this issue.

In response to genotoxic stress HIPK2 phosphorylates and activates the apoptotic program through interaction with diverse downstream targets including tumor suppressor p53 [7]. In addition, HIPK2 binds and phosphorylates a large number of targets, including signal transducers, transcription factors, epigenetic regulators, and ubiquitin ligases, regulating gene expression [39]. Therefore, besides alleviating transcription repression, HIPK2 elimination will also affect HIPK2-mediated p53 phosphorylation and activation. Accordingly, HG condition significantly reduced HIPK2-induced p53Ser46 phosphorylation, while a HIPK2 degradation-resistant mutant (K1182) failed to do so (Figure 5). As phosphorylation of p53Ser46 is essential for the induction of cell death [7], impaired HIPK2-mediated p53 modification will deteriorate chemotherapy-induced apoptosis in HG condition. In agreement, we previously found that HG impairs the cytotoxic effect of drugs in part through reduction of p53Ser46 apoptotic activity [23] and, as a proof of principle, preliminary data showed that in the early response to chemotherapeutic drugs HIPK2 protein failed to be stabilized and rather underwent downregulation (data not shown). However, why drug treatment was not protecting HIPK2 from HG-induced protein degradation needs to be elucidated.

Interestingly, here we noticed that HIPK2 degradation by HG could be alleviated by switching back cell culture from HG to low glucose condition (Figure 1D). Although a longer time course was not presented, our data suggest that lowering glycemic levels might be a valuable strategy to keep the HIPK2/p53 apoptotic axis functional. The translational potential of our finding could be also achieved by interfering with the mechanisms leading to HIPK2 downregulation. As a proof of principle, we showed here that culturing cells with hyperglycemic sera derived from patients could indeed reduce HIPK2 levels, compared to cells cultured with normo-glycemic sera and that the use of CL-A counteracted the degradative effect. In addition, we recently showed that ZnCl_2_ supplementation, by inhibiting the glycolytic pathway, efficiently restores the drug-induced cancer cell death impaired by HG [**25**]. Moreover, ZnCl_2_ has been also shown to inhibit HIF-1 activity and restore the HIPK2-induced p53 apoptotic activity [31, 36, 56]. We previously showed the beneficial effect of zinc supplementation in combination with chemotherapy [57–59] and here our findings further suggest that combination treatments with chemotherapeutic drugs and ZnCl_2_ may sustain the drug cytotoxic effect even in metabolic conditions that negatively affect apoptotic/chemoresistant pathways. Taken together, our results suggest that HG may play a critical role in reducing the apoptotic response in cancer cells by promoting HIPK2 degradation. As HIPK2 is a tumor suppressor and mediator of DNA damage-induced apoptosis, our results propose a new measure to improve the efficiency of genotoxic cancer therapies by interfering with the HG-induced pathways leading to HIPK2 degradation.

## MATERIALS AND METHODS

### Cell culture and reagents

Human colon cancer RKO and HCT116 and human embryo kidney (HEK)-293 cell lines were routinely cultured in Dulbecco modified Eagle's medium (DMEM) (Life Technology-Invitrogen, Carlsbad, CA, USA) containing 1 g/L D-glucose (low glucose - LG) supplemented with 10% heat-inactivated foetal bovine serum (FBS) (GIBCO-BRL, Grand Island, NY, USA) plus 100 units/ml penicillin/streptomycin and glutamine in 5% CO_2_ humidified incubator at 37°C.

For high glucose (HG) treatment, 24 h after plating cells were switched from LG medium to DMEM containing 4.5 g/L D-glucose (Life Technology-Invitrogen) supplemented with 2% FBS for 24 h, as previously reported [23, 27]. Human sera were obtained from type 2 diabetes patients (glycemia ≥ 300 mg/dl) or from healthy donors (glycemia ≤ 90 mg/dl) and used at 20% concentration to culture cells, as previously described [29].

Proteasome inhibitor MG132 (SIGMA-Aldrich, St. Louise, MO, USA) was used at 10 μM final concentration for 4 h, as previously reported [55]; phosphatase inhibitors calyculin A (SIGMA-Aldrich) and Okadaic acid (Santa Cruz Biotechnology, CA, USA) were used, respectively, at 1 nM and 5 nM for 24 h, as previously reported [24, 28]; PP2A activator FTY720 (SIGMA-Aldrich) was used at 5 μM for 16-24 h, as previously reported [30].

### Transient transfection, plasmids and luciferase assay

Cells were plated in 60 mm Petri dishes and, the day after, transfected with the cationic polymer LipofectaminePlus method (Life Technology-Invitrogen), according to the manufacturer's instructions. Expression vectors used were: empty-GFP, HIPK2-GFP [2], HIPK2^K1182R^-GFP (MDM2-degradation resistant) [14] and the vector encoding the dominant negative form of HIF-1α without DNA binding domain and transactivation domain (pCEP4-HIF-1αDN) [35, 36] (kindly provided by B.H. Jiang, Nanjing Medical University, China).

For p53 transcriptional activity, a luciferase assay was performed by co-transfecting cells with HIPK2-GFP or HIPK2^K1182R^-GFP expression vectors along with the luciferase reporter gene driven by the p53Ser46-dependent promoter p53AIP1-luc vector (kindly provided by H. Arakawa, National Cancer Center, Tokyo, Japan). For HIF1 transcriptional activity, a luciferase assay was performed by co-transfecting cells with the luciferase reporter gene driven by human erythropoietin enhancer region containing a functional HIF-1 binding site (pEpoE-luc) [33] (kindly provided by L. Eric Huang, N.C.I., N.I.H, Bethesda, MA, USA) with increasing amount of pCEP4-HIF-1αDN, as previously reported [33]. Transfection efficiency was normalized with a co-transfected β-galactosidase plasmid. Luciferase activity was assayed on whole cell extract and the luciferase values were normalized to β-galactosidase activity and protein content and expressed as relative luciferase unit (RLU).

### Immunocytochemistry

For immunofluorescence analysis, cells were transfected with HIPK2-GFP, HIPK2^K1182R^-GFP or empty-GFP vectors and, after transfection, trypsinized and re-plated on coverslips in low glucose medium. Twenty-four hours later, cells were switched to HG condition for additional 24 h. Cells were then washed twice with 1 x PBS and fixed in 4% formaldehyde at room temperature for 20 min. Cells were washed twice with 1 x PBS and then chromosomal DNA was stained with DAPI. GFP immunofluorescence was visualized by a Nikon Eclipse Ti-U fluorescence microscope (Nikon).

### Clonogenic survival assay

For survival assay, 2×10^5^ cells were plated on 60-mm dishes and transfected with HIPK2-GFP, K1182-GFP or empty-GFP vectors; 24 h later cells were switched to HG condition and death-resistant cells were stained with crystal violet 1 week later.

### PI staining

Apoptosis was quantified by cytofluorimetric analysis after staining cells with non-vital dye propidium iodide (PI) (Immunological Sciences, Rome, Italy), following the manufacturer's instruction. Briefly, cells floating were collected by centrifugation and pooled with adherent cells recovered from the plates, fixed in 80% ethanol, and stained in a PBS solution containing PI (62.5 mg/ml; Sigma-Aldrich) and RNase A Q10 (1.125 mg/ml; Sigma-Aldrich). Samples were analyzed with a FACScan instrument (Becton Dickinson Europe Holdings SAS - Le Pont De Claix, France) and the percentage of cells in subG1 compartment was calculated using ModFit LT software (Becton Dickinson). About 30 000 events were acquired and gated using forward scatter and side scatter to exclude cell debris.

### RNA extraction and semi-quantitative reverse transcription (RT)-PCR analysis

Cells were harvested in TRIzol Reagent and mRNA was isolated following the manufacturer's instructions (Life Technology-Invitrogen). The first strand cDNA was synthesized from 2 μg of total RNA with MuLV reverse transcriptase kit (Applied Biosystems, Foster City, CA, USA). Semi-quantitative Reverse-Transcribed (RT)-PCR was carried out by using Hot-Master Taq polymerase (Eppendorf, Milan, Italy) with 2 μl cDNA reaction and genes specific oligonucleotides under conditions of linear amplification. PCR products were run on 2% agarose gels and visualized with ethidium bromide. The housekeeping 28S gene, used as internal standard, was amplified from the same cDNA reaction mixture.

### Western blotting and immunoprecipitation

Total cell extracts were prepared by incubation in lysisbuffer (50 mM Tris-HCl, pH 7.5, 150 mM NaCl, 5 mM EDTA, pH 8.0, 150 mM KCl,1 mM dithiothreitol, 1% Nonidet P-40) and a mix of protease and phosphataseinhibitors (Roche, Indianapolis, IN, USA) on ice for 30 min. Cells were spun at 15000 × g for 20 min to remove debris and collect the supernatant. Protein concentration was then determined using BCA Protein Assay kit (BioRad, Hercules, CA, USA). Samples were denatured in SDS sample buffer. Total cell extracts (20–60 μg protein/lane) were resolved by 9–18% SDS polyacrylamide gel electrophoresis and transferred to polyvinylidene difluoride (PVDF) membranes (Merck Millipore, Billerica, MA, USA). Unspecific binding sites were blocked by incubating membranes for 1 h in 0.05% Tween-20 (v/v in TBS) supplemented with 5% non-fat powdered milk or bovine serum albumin (SIGMA-Aldrich), followed by overnight incubation with the following primary antibodies: rabbit polyclonal anti-HIPK2 (Abcam, Cambridge, UK), mouse monoclonal anti-GFP (Roche), rabbit polyclonal anti-GLUT-1 (H-43), rabbit polyclonal phospho-Ser46 (Santa Cruz Biotechnology), and rabbit polyclonal anti-PP2A-Cα (N-25) (Santa Cruz Biotechnology). Primary antibodies were detected with appropriate anti-immunoglobulin-G-horseradish peroxidase secondary antibodies (BioRad). Enzymatic signals were visualized using chemiluminescence (ECL Detection system, Amersham GE Healthcare, Milan, Italy), according to the manufacturer's protocol. Equal lane loading was monitored by probing membranes with antibodies specific for mouse monoclonal β-actin (Calbiochem, San Diego, CA, USA). Densitometry was performed with ImageJ software and relative band intensity normalized to β-actin and quantified with respect to controls set to 1.0.

### siRNA interference

Cells were plated at semiconfluence in 35-mm Petri dishes and, the day after, transfected with siRNA-control (si-ctr), si-PP2A-Cα or si-Siah2 (Santa Cruz Biotechnology) following the manufacturer's instructions. PP2A silencing was evaluated 48 h after transfection by western immunoblotting while Siah2 silencing was evaluated 48 h after transfection by RT-PCR analysis.

### Statistical analysis

Each experiment, unless differently specified, was performed at least three times. Results are reported as means ± standard deviation (S.D.). Statistical significance was determined using Student's t-tests for two sample comparisons and one-way ANOVA analysis for three or more sample comparisons. A value of p ≤ 0.05 was considered statistically significant.

## SUPPLEMENTARY MATERIAL


