# Supplementary material for: Hyperglycemia triggers HIPK2 protein degradation
Source: Oncotarget. 2016 Nov 25;8(1):1190–203. doi: 10.18632/oncotarget.13595 (PMC5352047; doi:10.18632/oncotarget.13595)
Supplement: Supplementary file 1 [file oncotarget-08-1190-s001.pdf]

Hyperglycemia triggers HIPK2 protein degradation

SUPPLEMENTARY MATERIAL

Immunoprecipitation

For phosphorylated HIPK2 immunoprecipitation, HEK-293 cells were co-transfected with HIPK2-GFP vector and, after transfection, switched to low and HG condition for 24 h. For preparation of cell lysates, cells were harvested and washed once with 1 x PBS; cell pellets were then incubated at 4°C for 30 min in lysis buffer (20 mM Hepes, 300 mM NaCl, 5 mM EDTA (pH 8.0), 10% glycerol, 1% NP-40) and a mix of protease and phosphatase inhibitors (Roche). Cells were spun at 15000 × g for 20 min to remove debris and collect the supernatant. The supernatants were diluted in lysis buffer and 200 µg cell lysates from LG condition, or 600 µg of cell lysates from HG condition were incubated with anti-GFP antibody pre-adsorbed to protein G-Sepharose beads (Roche), rocking at 4°C for 2 h. The beads were then washed four times with lysis buffer supplemented with protease inhibitors, and the immunoprecipitates were eluted by boiling in Laemmli buffer and resolved by 9–18% SDS-PAGE. Phosphorylated immunoprecipitated HIPK2-GFP was detected by western blotting using rabbit polyclonal anti p-Ser/Thr (Cell Signaling, Danvers, MA, USA) antibody as a primary antibody. Primary antibodies were detected with appropriate anti-immunoglobulin-G-horseradish peroxidase secondary antibodies (BioRad). Enzymatic signals were visualized using chemiluminescence (ECL Detection system, Amersham GE Healthcare, Milan, Italy), according to the manufacturer's protocol. The densitiy of the bands in the treated samples relative to that of control was analyzed and quantified by ScnImage software.

SUPPLEMENTARY FIGURES

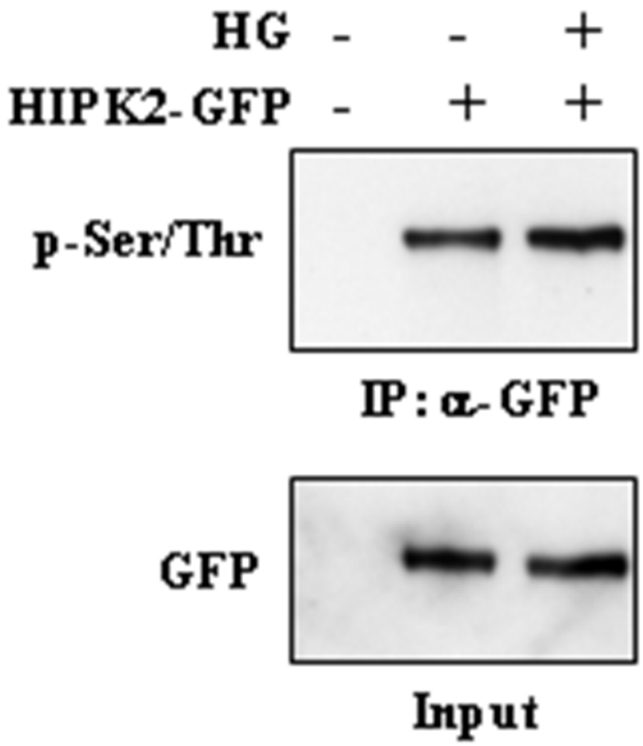

Supplementary Figure S1: Ser/Thr HIPK2 phosphorylation in normal and HG condition.
